# Pharmaceutical companies information and antibiotic prescription patterns: A follow-up study in Spanish primary care

**DOI:** 10.1371/journal.pone.0221326

**Published:** 2019-08-22

**Authors:** Iria Fernández-Álvarez, Maruxa Zapata-Cachafeiro, Juan Vázquez-Lago, Paula López-Vázquez, María Piñeiro-Lamas, Raquel García Rodríguez, Adolfo Figueiras, on behalf of the GREPHEPI Group

**Affiliations:** 1 Preventive Medicine Service, Santiago de Compostela University Teaching Hospital, Santiago de Compostela, Spain; 2 Department of Preventive Medicine and Public Health, University of Santiago de Compostela, Santiago de Compostela, Spain; 3 Consortium for Biomedical Research in Epidemiology & Public Health (*CIBER en Epidemiología y Salud Pública/CIBERESP*), Santiago de Compostela, Spain; 4 Health Research Institute of Santiago de Compostela (*IDIS*), Santiago de Compostela, Spain; 5 Preventive Medicine Service, A Coruña University Teaching Hospital, Coruña, Spain; Rabin Medical Center, Beilinson Hospital, ISRAEL

## Abstract

**Objectives:**

To assess the impact of sources of drug information on antibiotic prescribing patterns (quantity and quality) among primary care physicians.

**Methods:**

We conducted a cohort study on primary care physicians who were actively engaged in medical practice in 2010 in a region in north-west Spain (Galicia), fulfilling inclusion criteria (n = 2100). As the independent variable, we took the perceived utility of 6 sources of information on antibiotics, as measured by the validated KAAR-11 questionnaire. As dependent variables, we used: (1) a quality indicator (appropriate quality, defined as any case where 6 of the 12 indicators proposed by the European Surveillance of Antimicrobial Consumption Network [ESAC-Net] were better than the mean values for Spain); and, (2) a quantity indicator (high prescribing), defined as any case where defined daily doses (DDD) per 1 000 inhabitants per day of antibacterials for systemic use were higher than the mean values for Spain. The adjusted odds ratio for a change in the interquartile range (IqOR) for each sources of information on antibiotics was calculated using Generalized Linear Mixed Models.

**Results:**

The questionnaire response rate was 68%. Greater perceived utility of pharmaceutical sales representatives increases the risk of having high prescribing (1/IqOR = 2.50 [95%CI: 1.63–3.66]) and reduces the probability of having appropriate quality (1/IqOR = 2.28 [95%CI: 1.77–3.01]). Greater perceived utility of clinical guidelines increases the probability of having appropriate quality (1/IqOR = 1.25 [95%CI: 1.02–1.54]) and reduces the probability of high prescribing (1/IqOR = 1.25 [95%CI: 1.02–1.54]).

**Conclusions:**

Sources of information on antibiotics are an important determinant of the quantity and quality of antibiotic prescribing in primary care. Commercial sources of information influence prescribing negatively, and clinical guidelines are associated with better indicators.

## Introduction

Misuse of antibiotics is the principal cause of the problem of resistance. [1,2] Notwithstanding the strategies developed to improve prescribing [3,4] antibiotic use continues to grow. [5]Around 90% of all antibiotic prescriptions are issued in primary care, [6–8] where an appreciable gap has been observed between clinical guidelines and practice. [9] The factors that affect misprescribing in community settings are both numerous and inter-related, [10,11] and predominant among them are the sources of information through which physicians acquire information.

The sources of information used by physicians have been described as one of the main determinants of drug prescribing quality, [12–15] and there is evidence to show the important role played by the pharmaceutical industry: indeed, exposure to pharmaceutical company information has been seen to be associated with higher prescribing, higher costs and lower prescribing quality. [13–15]

However, there are few studies that specifically evaluate the influence of the sources of information used by physicians on antibiotic prescribing, [15–19] despite the fact that, in such a case, any misprescription, in addition to raising the risk of adverse events and healthcare costs, would also increase the development of resistance. [20]

Accordingly, this paper sought to evaluate the influence of the perceived utility of drug information sources, on drug quality and quantity. To this end, we used the KAAR-11 questionnaire, [21] which was developed by our team on the basis of focal group studies, [22] and has already been used in previous studies [23,24] to identify attitudes and knowledge associated with misprescription of antibiotics.

## Methods

### Study setting

Our study was conducted in Galicia. Galicia is a region in north-west Spain with a population of 2.7 million, 24.3% of whom are over 65 years of age. Population density is 91,4 habitants/m2 and decreases as one moves inland from the coast, so distances to a public health centre tend to increase. [25] Practically all the population is covered by the Spanish National Health System (NHS). The NHS is almost fully funded by taxes and provision of all health services, other than pharmaceuticals, is free of charge at the point of delivery. Prior to 2012, the NHS afforded universal coverage, and pharmaceutical services were co-financed by outpatients, with pensioners and their beneficiaries being exempt from co-payment, and non-pensioners and their beneficiaries paying 40% of the retail price. Medication may only be dispensed by community or hospital pharmacies, and for some types of medication, e.g., antibiotics, a physician's prescription is compulsory; even so, the dispensing of drugs without a prescription continues to exist in Spain. [26,27] In Galicia, outpatient care is organised in two systems, namely: healthcare or primary care centres, where medical care is given by appointment and each physician is assigned a fixed population quota; and 24-hour emergency outpatient centres, where there are no population quotas assigned to each physician, but emergency care is instead given on demand to whatever proportion of the population that requires it.

In Spain, relations between the pharmaceutical industry and the healthcare system are partially regulated by domestic legislation; [28,29] legislative gaps are covered by the industry, and self-regulation predominates in the form of a Good Practice Code. [30] However, the degree of compliance with these rules and regulations is not optimal. [31–33]

### Study design and population

A cohort study covering all primary care physicians working for the NHS in Galicia in 2010 (N = 3675) was carried out. From those the following were excluded: physicians exclusively assigned to emergency services; medical residents in training; and temporary staff. [23] Physicians exclusively assigned to emergency services were excluded, since they do not have a designated number of listed patients, thus rendering it impossible to calculate DID (Defined daily dose -DDD- per 1000 Inhabitants per Day) indicators that require the number of patients attended as their denominator.

### Measure

All physicians who fulfilled the inclusion criteria were sent a letter describing the study, a questionnaire on antibiotic prescribing, a prepaid self-addressed envelope for returning the completed questionnaire, as well as a small gift (ballpoint pen or pencil). In cases where there was no response to the questionnaire, this was re-sent a maximum of 4 times.

The validated KAAR-11 (**K**nowledge and **A**ttitudes regarding **A**ntibiotics and **R**esistance) questionnaire was used: further details about the development and validation of the KAAR-11 questionnaire can be found elsewhere. [21] This questionnaire evaluates: (i) the perceived utility of sources of information (clinical practice guidelines, documentation from the pharmaceutical industry, pharmaceutical industry training (courses, sessions, congresses … organized specifically by the pharmaceutical industry), medical representatives (or pharmaceutical sales representatives), previous clinical experience and other specialist) in the management of upper respiratory tract infections; (ii) healthcare criteria which may influence prescribing (number of patients, time devoted to each patient, and whether or not physicians work on-call duty shifts); and (iii) knowledge and attitudes regarding antibiotics and resistance. The results were assessed using a continuous, horizontal, visual analogue scale (VAS), scored in a range from 0 (total disagreement) to 10 (total agreement).

### Follow-up and outcome measures

The NHS provided monthly online prescription records for physicians from January 2008 to December 2010. Drug-indication information was, however, unavailable.

To assess the quantity and quality of antibiotic prescriptions (dependent variables) we calculated the 12 indicators proposed by the European Surveillance of Antimicrobial Consumption (ESAC), [34] validated by Coenen et al, [35] which we then used to construct the dichotomous dependent variables, namely, the quantity and quality of prescriptions. In the case of the quantity of prescriptions we used indicator J01_DID (consumption of antibacterials for systemic use expressed in defined daily doses/1000 inhabitants/day). Appropriate prescribing quantity (lower quantity) was assumed to exist in any case where the value of this indicator was better than the reference value for Spain. [23] Prescribing quality was deemed to be appropriate (Appropriate Quality Prescription of Antibiotics: AQPA) in any case where half (6/12) or more of the annual ESAC indicators for each physician were better than the reference values for Spain (see Apendix 1). We have selected these cut-points to avoid their opportunistic selection. [23,24] Moreover, we considered the characteristics of the primary care centers that could act as potentially confusing covariates, in addition to the care criteria that were collected in the KAAR-11 questionnaire: rural/urban primary care center; interior/coast primary care center; availability of extra-hospital emergency services; center with/without residency training; center with specialties; and distance from the primary care center to the nearest hospital.

### Statistical analysis

Generalized linear mixed models were applied to statistical analysis. To construct the models, we used the quantity (J01_DID ESAC indicator) and the quality of prescription (AQPA) as dependents variables, with individual observations (per year and physician) as level 1 and physicians as level 2; random effects were considered among physicians using R (R Foundation for Statistical Computing). In view of the fact that the response variables were dichotomous, these models were fitted with the binomial family, using the lme4 package for the R free software environment for statistical computing. [36] To construct the models, we first performed a bivariate analysis, and then selected all exposure and potential confounding variables having a P-value lower than 0.2. Second, the variables so selected were then studied in a multivariate analysis. The variables with the highest level of statistical significance were successively eliminated, provided that the coefficients of the principal variables of exposure changed by no more than 10% and improved the Schwartz’s Bayesian Information Criterion, [37] until the most appropriate model had been obtained.

To take into account the scale of independent variables and their distribution among the study subjects, we calculated the interquartile odds ratio (Iq OR), which is based on an incremental exposure corresponding to the interquartile range on the scale of the perceived utility of the source of information measures. [38] To express the results, we applied the following three-step procedure:

- firstly, we calculated the odds ratios (ORs) with their 95% confidence intervals **for the continuous variable**, which indicates the increase/decrease in the probability of having appropriate prescribing quantity or quality for an increase of 1 unit on the continuous VAS (scale range 0–10) that measured the perceived utility of sources of information;
- secondly, we calculated the Iq OR, by **multiplying the IQR (Interquartile Range)** by the OR; and,
- thirdly, in any case where the OR was lower than 1, we calculated the inverse of the Iq OR (1/Iq OR), which can be interpreted as the increase in the probability of having appropriate prescribing quantity or quality when scale values decrease from the 75^th^ to the 25^th^ percentile.

### Ethics and confidentiality

The study was approved by the Galician Ethics Committee (code number 2007/107). The physicians included in the study were informed through a letter, as described in the section measures. The physicians accepted to participate in the study if they answered the questionnaire. To ensure confidentiality, once the data needed to obtain the prescription indicators had been linked to the results of the questionnaires, they were furnished by the Galician NHS in an anonymised format such that no indicator could be related to a specific professional.

## Results

A total of 2100 (57.1%) physicians fulfilled the inclusion criteria, with a 68% (n = 1428) response rate to the postal questionnaire. The indicator values for responders and non-responders were very similar. [23] In terms of quantity, 21.2% of responders registered better prescription values (indicator J01_DID) than the Spanish mean, and 66.1% registered AQPA.

Table 1 shows that a higher degree of the management of upper respiratory tract infections deriving from information sources from pharmaceutical companies was associated with a higher probability of prescribing more antibiotics, namely, 1/IqOR = 2.50 (95%CI: 1.63–3.66, *p*<0.001) for pharmaceutical sales representatives and 2.09 (95%CI: 1.70–2.87, *p*<0.001) for pharmaceutical documentation. In contrast, the management of upper respiratory tract infections deriving from clinical guidelines was associated with a lower probability of antibiotic prescribing: IqOR = 1.25; 95%CI: 1.02–1.54, *p* = 0.031).

**Table 1:** Association between information sources and antibiotic prescribing in terms of quantity (OR of a physician registering values better than the Spanish reference for ESAC indicator J01_DID,^a^ on comparing the 25^th^ to the 75^th^ percentile of each source of information).

	Percentile			Quantity		
*Source of information*^b^	25	50	75	Iq OR (95% CI)	Inverse Iq OR (95% CI)	*p* value
Clinical Practice Guidelines	7.5	9	9.5	1.25 (1.02–1.54)	—	0.031
Documentation of Pharmaceutical Industry	2	4	5.5	—	2.09 (1.70–2.87)	<0.001
Pharmaceutical Industry Training	2.5	5	6	—	1.45 (0.93–1.15)	0.188
Medical Representatives	1.5	3	5	—	2.50 (1.63–3.66)	<0.001
Previous clinical experience	7	8	9.5	—	1.27 (0.77–2.12)	0.348
Other specialists	7	8.5	9.5	—	1.03 (0.93–1.23)	0.888

Abbreviations. Iq OR: interquartile odds ratio; CI: confidence interval; DID: DDDs (assumed average maintenance dose per day for a drug used for its main indication in adults) per 1000 inhabitants per day.

a J01_DID: consumption of antibacterials for systemic use expressed in DID.

b Measured using a continuous, horizontal, visual analogue scale. Recorded answers were scored in a range from zero (total disagreement) to ten (total agreement).

Table 2 shows that a higher degree of the management of upper respiratory tract infections deriving from sources of information from the pharmaceutical industry was associated with worse prescribing *quality*, namely, 1/IqOR = 2.28 (95%CI: 1.77–3.01, *p*<0.001) for pharmaceutical sales representatives, 2.28 (95%CI: 1.70–3.01, *p*<0.001) for pharmaceutical industry courses, and 2.09 (95%CI: 1.63–2.74, *p*<0.001) for pharmaceutical documentation. A higher degree of management of upper respiratory tract infections deriving from clinical guidelines was associated with better quality but with effect magnitudes lower than those deriving from commercial guidelines: IqOR = 1.25 (95%CI: 1.02–1.54, *p* = 0.026).

**Table 2:** Association between information sources and antibiotic prescribing in terms of quality (OR of a physician showing *Appropriate Quality Prescription of Antibiotics*,^a^ on comparing the 25^th^ to the 75^th^ percentile of each source of information).

	Quality		
*Source of information*^b^	Iq OR (95% CI)	Inverse Iq OR (95% CI)	*p* value
Clinical Practice Guidelines	1.25 (1.02–1.54)	—	0.026
Documentation of Pharmaceutical Industry	—	2.09 (1.63–2.74)	<0.001
Pharmaceutical Industry Training	—	2.28 (1.70–3.01)	<0.001
Medical Representatives	—	2.28 (1.77–3.01)	<0.001
Previous clinical experience	—	2.35 (1.80–3.05)	<0.001
Other specialists	—	1.23 (1.00–1.55)	0.057

Abbreviations. Iq OR: interquartile odds ratio; CI: confidence interval; DID: DDDs (assumed average maintenance dose per day for a drug used for its main indication in adults) per 1000 inhabitants per day.

a Appropriate quality prescription of antibiotics (AQPA): half or more ESAC indicator values better than the reference values for Spain.

b Measured using a continuous, horizontal, visual analogue scale. Recorded answers were scored in a range from zero (total disagreement) to ten (total agreement).

## Discussion

The results of this large-sized cohort study show that sources of information on antibiotics are an important determinant of the quantity and quality of antibiotic prescribing in primary care. Whereas commercial sources of information influence prescribing negatively, clinical guidelines are the sole resource associated with better indicators, albeit with lower effect magnitudes.

The considerable influence exerted by the pharmaceutical industry on antibiotic misprescribing (with effect magnitudes of more than twofold) is a reflection of the important role that it plays in antibiotic prescribing. These results are consistent, both with research results on the effect of pharmaceutical company information on global prescribing habits, and with the volume of resources that the industry allocates to the promotion of medications. [19, 39] Despite the pharmaceutical sector’s proposals for self-regulation and the reduction in the number of pharmaceutical sales representatives after the 2010 economic crisis, the majority of primary care physicians regularly receive pharmaceutical sales representatives and accept their gifts. [40] Moreover, regulations governing the relations between the industry and the healthcare system are frequently infringed. [41] Influence on prescribers is based on **psychological mechanisms** that determine clinical decision-making. Physicians display contradictions in their relationship with the industry, justifying it and considering that it does not affect them on an individual basis, while at the same time regarding it as affecting their colleagues. [42] According to some authors, they develop strategies to resolve cognitive dissonance, which entail conserving a self-image of independence and credibility without dispensing with their omnipresent interactions with the industry. [43]

The high effect magnitude encountered by us could also be due to the fact that antibiotics are a group of medical drugs with specific characteristics which can be used for their promotion and could favour their over/misprescribed: (1) promotion based on effectiveness rather than appropriateness; (2) favouring the short-term view of the *individual* patient to the detriment of a longer-term population-based stance; and/or, (3) advocating prescribing in the face of diagnostic uncertainty and/or fear of the consequences of not prescribing. [23] The perceived utility of clinical guidelines is the only resource associated with better prescribing, albeit with a much lower magnitude (IqOR = 1.25) than that of commercial sources, which indicates that these would seem to have less influence. This finding is consistent with the underuse of guidelines [44,45] and the gap between evidence-based recommendations and clinical reality. [46–48] Even though there is evidence to show that clinical guidelines could be biased due to authors’ conflicts of interest with pharmaceutical companies, [49] this fact is probably less worrying in the case of antibiotics. Something that also has to be borne in mind is that public concern about antimicrobial resistance tends to favour greater control of rational antibiotic use, which is reflected in more independent guidelines. [50–52]

Our study has a series of strengths, the chief of which is the use of the first fully-validated questionnaire, [21] which ensures that our findings are based, not on simulations, but on real physician-based prescription data. [53] Furthermore, the use of ESAC indicators [35] guarantees the absence of opportunism in the choice of the most favourable indicators. In addition, the use of these indicators favours the comparability of results at European level.

Some limitations must also be considered. Firstly, there could be a tendency on the part of physicians to answer in way that they perceive as being more socially acceptable (social desirability bias). [54] However, this would affect the *absolute scores* but not the calculation of effect measures (IqOR). Secondly, while a possible non-response bias might exist, it has to be said that, not only was the response rate (68%) higher than that obtained in studies with postal questionnaires, [55] but there were hardly any differences between responders and non-responders. [23] Thirdly, even though data on indications were not available to us, we nevertheless feel that, due the large size of the number of registered patients per physician (median 1329) and the analysis being adjusted for potential confounding factors, differences in quality and quantity indicators among physicians cannot be attributed to variations in patient morbidity. Fourth, It is possible that there may be differences in the patient mix in a specific primary care practice (e.g., more elderly patients, lower or higher area served) but we feel that such differences should not be very great, given the large size of physicians’quotas (n>1000) and the fact that most of the indicators are ratios or proportions relative to the size of the population attended (DHD, District Health Department) or, ratios or proportions whereby the indicators are automatically adjusted. Should there be differences between physicians’ quotas which might affect outcomes (quantity and quality of prescriptions), we feel that this would not be associated -positively or negatively- with the perceived utility of physicians’ sources of information. This could cause a non-differential misclassification in the outcome (indicators), which would lead to an underestimate of the effect (and in turn towards the null hypothesis). [56] If, despite this potential underestimate, physicians’ commercial sources of information show an effect, it can be assumed that the effect would be greater still.

In view of the fact that there are local circumstances (organisation of the healthcare system, legislation of relations with the industry, physicians antibiotic culture, etc.) which can affect the results obtained and determine the applicability of our results to different healthcare systems, we feel that generalizability should be approached with caution, though there are numerous studies which have described the **pharmaceutical industry ‘s** influence on the healthcare system [57] and drug prescribing. [58–62] Moreover, it is possible that in medical circles that are less regulated than Spain’s (e.g., some developing countries), the influence exerted by information provided by the industry on prescribing patterns could be even greater. [62,63].

In conclusion, improving antibiotic prescribing is a complex task that calls for multiple complementary approaches. Reducing the gaps between scientific evidence and clinical practice is one of the challenges that must be overcome to achieve this goal. Our results seem to support the need to reduce the effect of the industry and foster the use of more independent resources, such the adequate implementation of independent clinical guidelines, in order to rationalise antibiotic prescribing.

## Supporting information

S1 File Data. This is the data file.
(RAR)

S1 Appendix ESAC indicators for Spain 2009. (PDF)
