# Supplementary material for: Pharmaceutical companies information and antibiotic prescription patterns: A follow-up study in Spanish primary care
Source: PLoS One. 2019 Aug 22;14(8):e0221326. doi: 10.1371/journal.pone.0221326 (PMC6706057; doi:10.1371/journal.pone.0221326)
Supplement: S1 Appendix — (PDF) [file pone.0221326.s002.pdf]

Quality indicators for antibiotic consumption in the community (primary care sector) in Europe 2009

Country	Consumption					Relative consumption				Broad/narrow	Seasonal variation	
	J01*	J01C	J01D	J01F	J01M	J01CE_%	J01CR_%	J01DD+DE_%	J01MA_%	J01_B/N	J01_SV	J01M_SV
Austria	15.49	6.64	1.80	3.93	1.33	6.4%	29.1%	5.1%	8.6%	7.39	137.0%	116.9%
Belgium	27.55	15.13	1.82	2.99	2.61	0.4%	32.3%	<0.1%	9.5%	40.59	133.6%	118.2%
Bulgaria	18.35	8.40	2.30	2.96	1.97	2.0%	14.6%	0.9%	10.7%	5.97	-	-
Croatia												
Cyprus**	34.45	16.01	6.45	3.98	4.13	0.3%	29.3%	1.7%	12.0%	26.93	-	-
Czech Republic	18.44	7.73	1.55	3.66	1.27	11.2%	21.1%	0.4%	6.9%	4.06	119.1%	109.1%
Denmark	15.58	9.86	0.03	2.26	0.52	32.1%	2.6%	<0.1%	3.3%	0.37	118.2%	106.6%
Estonia	11.06	4.37	0.83	2.09	0.79	2.2%	10.8%	<0.1%	7.1%	7.86	131.2%	104.4%
Finland	17.89	6.08	2.33	1.46	0.87	7.7%	6.9%	<0.1%	4.9%	0.73	112.3%	106.8%
France	29.58	16.08	2.92	4.15	2.00	0.5%	21.9%	6.4%	6.5%	42.76	-	-
Germany	14.90	4.27	2.39	2.51	1.48	5.7%	2.0%	3.4%	9.9%	3.98	146.1%	131.5%
Greece**	38.63	12.89	8.68	11.53	2.63	1.9%	13.7%	0.8%	6.8%	31.72	132.5%	103.2%
Hungary	15.98	7.06	1.98	3.00	1.79	4.2%	28.8%	2.4%	11.0%	12.95	157.4%	125.1%
Iceland	19.35	10.41	0.30	1.15	0.55	12.1%	18.3%		2.9%	1.67	113.5%	105.4%
Ireland	20.76	10.66	1.33	3.79	0.94	4.1%	26.5%	0.5%	4.5%	5.44	118.8%	104.1%
Italy	28.66	15.18	2.78	5.33	3.61	<0.1%	34.3%	7.2%	12.1%	99.28	-	-
Latvia	10.90	4.99	0.45	0.90	0.89	1.5%	12.5%	0.5%	7.7%	6.23	133.5%	119.6%
Liechtenstein												
Lithuania**	19.45	10.05	1.27	1.93	1.23	4.6%	8.8%	0.4%	5.8%	2.59	-	-
Luxembourg	28.19	13.47	4.33	3.87	2.81	0.3%	29.9%	<0.1%	10.0%	33.84	141.9%	125.3%
Malta	21.59	9.08	5.50	3.89	1.66	0.1%	36.4%	0.8%	7.7%	149.49	-	-
Netherlands	11.35	4.48	0.04	1.48	0.89	3.4%	16.0%	<0.1%	7.7%	6.20	118.0%	102.5%
Norway	15.23	6.59	0.13	1.68	0.51	23.9%	<0.1%	<0.1%	3.3%	0.19	-	-
Poland	23.59	10.68	2.89	3.88	1.25	0.6%	20.9%	<0.1%	5.3%	36.28	-	-
Portugal	22.94	12.00	1.96	3.83	3.04	<0.1%	39.2%	1.7%	13.3%	23.24	127.5%	107.4%
Romania	10.19	4.31	2.47	1.84	1.26	1.6%	23.6%	1.0%	12.3%	6.10	-	-
Slovakia	23.78	9.56	4.12	6.09	2.03	7.8%	22.7%	2.3%	8.6%	7.39	135.1%	110.3%
Slovenia	14.31	9.51	0.42	2.23	1.08	13.6%	28.5%	0.8%	7.5%	3.42	125.8%	109.8%
Spain	19.68	12.31	1.56	1.90	2.42	0.5%	38.7%	2.8%	12.0%	56.89	125.8%	117.3%
Sweden	14.08	7.00	0.24	0.76	0.79	27.6%	1.7%	0.2%	5.6%	0.17	111.6%	101.2%
United Kingdom	17.27	8.03	0.58	2.51	0.48	4.3%	6.4%	<0.1%	2.8%	0.84	117.2%	107.6%

* Denominator for relative consumption; ** Country provided only total care data
Total care includes data both from the hospital and community sectors and overestimates the figures when used for reporting for the community sector.
The quality indicators presented should not be used alone to evaluate the effect of programmes towards prudent use in the community. These indicators are not applicable for the hospital sector.

Quality indicators		
Consumption		
J01	J01_DID***	Consumption of antibacterials for systemic use (J01) expressed in DDD per 1000 inhabitants and per day
J01C	J01C_DID	Consumption of penicillins (J01C) expressed in DDD per 1000 inhabitants and per day
J01D	J01D_DID	Consumption of cephalosporins (J01D) expressed in DDD per 1000 inhabitants and per day
J01F	J01F_DID	Consumption of macrolides, lincosamides and streptogramins (J01F) expressed in DDD per 1000 inhabitants and per day
J01M	J01M_DID	Consumption of quinolones (J01M) expressed in DDD per 1000 inhabitants and per day
Relative consumption		
J01CE	J01CE_%	Consumption of beta-lactamase sensitive penicillins (J01CE) expressed as percentage of the total consumption of antibacterials for systemic use (J01)
J01CR	J01CR_%	Consumption of combination of penicillins, including beta-lactamase inhibitor (J01CR) expressed as percentage of the total consumption of antibacterials for systemic use (J01)
J01DD+DE	J01DD+DE_%	Consumption of third- and fourth-generation cephalosporins (J01(DD+DE)) expressed as percentage of the total consumption of antibacterials for systemic use (J01)
J01MA	J01MA_%	Consumption of fluoroquinolones (J01MA) expressed as percentage of the total consumption of antibacterials for systemic use (J01)
Broad/narrow		
J01	J01_B/N	Ratio of the consumption of broad-spectrum (J01(CR+DC+DD+(F-FA01))) to the consumption of narrow-spectrum penicillins, cephalosporins and macrolides (J01(CE+DB+FA01))
Seasonal variation		

J01	J01_SV	Seasonal variation of the total antibiotic consumption (J01)<br>Seasonal variation: Overuse in the winter quarters (January-March and October-December) compared with the summer quarters (April-June and July-September) of a 1-year period starting in July and ending the next calendar year in June, expressed as percentage: [DDD per 1000 inhabitants and per day (winter quarters)/DDD per 1000 inhabitants and per day (summer quarters)-1] x100
J01M	J01M_SV	Seasonal variation of quinolone consumption (J01M)

*** This column displays the original labels of the quality indicators as described in the article Coenen S, Ferech M, Haaijer-Ruskamp FM, et al. European Surveillance of Antimicrobial Consumption (ESAC): quality indicators for outpatient antibiotic use in Europe. published in Qual Saf Health Care 2007;16:440?445.

General comments for comparison of antimicrobial consumption data from different countries and/or years:
Total care includes data both from the hospital sector and community (primary care sector) and overestimates the figures when used for reporting for the community sector.

Some countries report reimbursement data that do not include consumption of antimicrobials obtained without prescription and other non-reimbursed courses.

For more information please see the report "Data source overview of antimicrobial consumption"

Finland: hospital sector data include consumption in remote primary health care centres and nursing homes, thus overestimating hospital consumption and underestimating community consumption compared to the other countries that report data from these sources as community consumption.

This report has been generated from ESAC-Net data submitted to TESSy, The European Surveillance System on 2015-01-12.
The report reflects the state of submissions in TESSy as of 2015-01-12 at 14:00
